# Enhancing the Antimicrobial Properties of Experimental Resin-Based Dental Composites through the Addition of Quaternary Ammonium Salts

**DOI:** 10.3390/jfb15080213

**Published:** 2024-07-30

**Authors:** Joanna Nowak, Maja Zalega, Witold Jakubowski, Monika Domarecka, Jerzy Sokołowski, Kinga Bociong

**Affiliations:** 1 University Laboratory of Materials Research, Medical University of Lodz, ul. Pomorska 251, 92-213 Lodz, Poland; 2 Department of General Dentistry, Medical University of Lodz, ul. Pomorska 251, 92-213 Lodz, Poland; 3 Division of Biophysics, Institute of Materials Science and Engineering, Lodz University of Technology, ul. Stefanowskiego 1/15, 90-924 Lodz, Poland

**Keywords:** dental composite, quaternary ammonium salts, DODAB, CTAB, anticaries

## Abstract

Secondary caries is one of the main reasons for dental filling replacement. There is a need to obtain dental restorative material that is able to act against caries-inducing microorganisms. This study explores the antimicrobial properties of cetyltrimethylammonium bromide (CTAB) or dimethyldioctadecylammonium bromide (DODAB)-modified photo-cured experimental dental composites against *Escherichia coli, Streptococcus mutans,* and *Candida albicans*. The antimicrobial activity against *Escherichia coli*, *Streptococcus mutans,* and *Candida albicans* was assessed by using an Accuri C6 flow cytofluorimeter, and then analyzed using BD CSampler software (1.0.264). Bacterial/yeast surface colonization was carried out by using an GX71 inverted-optics fluorescence microscope equipped with a DP 73 digital camera. For bactericidal surface analysis of each sample type, simultaneous standardization was performed using a positive control (live cells) and a negative control (dead cells). A positive correlation between the increasing concentration of CTAB or DODAB and the dead cell ratio of *Escherichia coli*, *Streptococcus mutans,* and *Candida albicans* was revealed. In particular, CTAB at a 2.0 wt% concentration exhibits superior efficiency against pathogens (65.0% dead cells of *Escherichia coli*, 73.9% dead cells of *Streptococcus mutans*, and 23.9% dead cells of *Candida albicans* after 60 min). However, *Candida albicans* is more resistant to used salts than bacteria. A CTAB- or DODAB-modified experimental dental composite exhibits antimicrobial potential against *Escherichia coli, Streptococcus mutans,* and *Candida albicans* after 10 and 60 min of incubation, and the antimicrobial efficiency increases with the wt% of QAS in the tested material.

## 1. Introduction

In modern dentistry, dental composites have become popular choices for restorative materials due to their excellent aesthetics and acceptable mechanical properties. To further develop this group of restorative materials, the World Health Organization (WHO) suggests conducting research focused on incorporating additives with antimicrobial properties into dental materials to prevent caries, especially secondary caries [1,2,3,4,5]. We can point out various groups of compounds that act against bacteria, viruses, and fungi in an oral environment, i.e., antibiotics (penicillin, tetracyclines, metronidazole, macrolides, clindamycin ), fluoride, chitosan, chlorhexidine (which is still the “gold standard” of anticaries agents), antimicrobial peptides and enzymes, remineralizing agents, metal and metal oxides, triclosan, quaternary ammonium compounds (QACs), and others [6,7,8,9,10,11,12]. Among these additives, quaternary ammonium salts (QASs) have shown great potential in inhibiting bacterial growth and in caries prevention in dental composites [6,13]. QASs are known as organic compounds containing a central nitrogen atom bound to four alkyl or aryl groups. The formula for QASs can be noted as N^+^R_1_R_2_R_3_R_4_X, where R may be a hydrogen atom, alkyl, or alkyl derivative group, and X most often denotes a halide anion like chloride or bromide, which are more effective than other anions. In polymers with QASs, direct copolymerization or post-polymerization with quaternary ammonium functional groups may occur. This leads to obtaining polymerizable quaternary ammonium methacrylates. These molecules possess a cationic head and hydrophobic tail, making them effective antimicrobial agents [4,9,10,11]. In dental materials, many QACs have been used, for instance:

- In dental resins—dimethylaminododecyl methacrylate (DMADDM) [3], urethane dimethacrylate monomer with two quaternary ammonium groups [14], and quaternary ammonium methacrylate monomers [15];
- In dental primers/adhesives: 12-methacryloylooxydodecylpiridinium bromide (MDPB) is used as an adhesive system in Clearfil Protect Bond^TM^ (Kuraray Co., Ltd., Tokyo, Japan) [11], DMADDM [16], and methacryloxyethyl cetyl dimethyl ammonium chloride (DMAE-CB) [17];
- In dental composites: 2-methacryloxyethyl hexadecyl methyl ammonium bromide (MAE-HB) [18], cetyltrimethylammonium bromide (CTAB), dimethyldioctadecylammonium bromide (DODAB) [2,4], CHX released from a dental composite reduces bacterial adhesion to the dental material without harmful effects on the oral environment [19];
- In denture-based acrylic resins: Poly 202063A [20];
- Quaternary ammonium micro-fillers and nanofillers, like quaternary ammonium poly(ethylenimine) nanoparticles (QPEI), quaternary ammonium silane-functionalized methacrylate, and quaternary ammonium silica (QASi) [11,21,22,23].

QASs are positively charged molecules that destroy negatively charged cell membranes. In the aftermath, the release of potassium ions from the cytoplasm (cytoplasmic leakage) and other important cytoplasmic components occurs, causing the death of pathogens. A complete QAS mode of action is not clearly understood as yet [4,9,10,11,24,25]. The antibacterial ability of QASs increases with the elongation of the alkyl moieties on the nitrogen atom, with the optimal length ranging from 10 to 18 carbon atoms in the alkyl chains (C-10 to C-18) [3,8,9,24,26,27]. It is worth noting that chains with 12 carbons are the most effective against yeasts and fungi, with 14 carbons being optimal for Gram-positive bacteria and 16 carbons for Gram-negative type [10]. 

**Figure 1:**
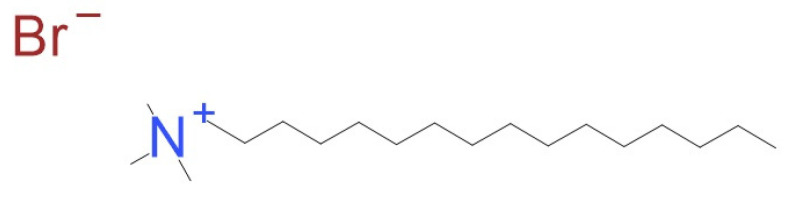
CTAB structure.

**Figure 2:**
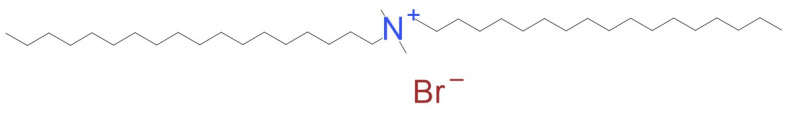
DODAB structure.

CTAB (Figure 1) is a cationic surfactant used to obtain mesoporous silica nanoparticles [28]. It also has antibacterial properties, i.e., against *Escherichia coli*, due to the indication of superoxide stress in bacteria cells, and it also penetrates the cell membrane, causing the leakage of essential components of bacteria cells [29,30]. DODAB (Figure 2) is a synthetic versatile lipid used as a surfactant or vaccine adjuvant, and it can create cationic membranes. It also may have potential applications in gene therapy and drug delivery. Due to the positively charged cationic small “head”, the DODAB membrane is stable and interacts well with negatively charged entities. Both CTAB and DODAB alter the bacteria cell surface charge, causing their death. DODAB is also known as an effective flocculant agent [31,32]. 

*Streptococcus mutans* and *Candida albicans* may develop acidic conditions that promote the demineralization of enamel and dentin, which may cause diseases like primary/secondary caries. An epidemiologic study has shown *Streptococcus mutans* as the most common pathogen isolated from dental plaque. It is most often used in antibacterial tests of dental materials as Gram-positive bacteria. It is the primary microorganism associated with dental caries. At the same time, *Escherichia coli* are common bacteria used to test Gram-negative types. *Candida albicans* is a fungus suitable for developing recurrent decay and candida-induced denture stomatitis. Strong interaction between *Candida albicans* and *Streptococcus mutans* significantly impacts caries development. This yeast can increase the virulence of *Streptococcus mutans* [5,6,9,12]. Although there is a lack of long-term research on the biocide activities of QACs, *Streptococcus mutans* could develop resistance to cationic antimicrobials. However, QAC-resistant strains mainly develop at sub-inhibitory concentrations of quaternary ammonium compounds [10].

The aim of this paper is to assess the antimicrobial and antifungal properties of experimental resin dental composite modified with cetyltrimethylammonium bromide (CTAB) or dodecyl dimethyldioctadecylammonium bromide (DODAB), depending on the additive amount. The null hypothesis is that there will be no differences between CTAB- and DODAB-modified composites and their microbial activities.

## 2. Materials and Methods

### 2.1. Chemicals and Reagents

The experimental dental composite resin matrix contained the following: 40 wt% bisphenol A glycerolate dimethacrylate (bis-GMA), 40 wt% diurethane dimethacrylate (UDMA), 10 wt% 2-hydroxyethyl methacrylate (HEMA), and 10 wt% triethylene glycol dimethacrylate (TEGDMA). Moreover, 0.4 wt% of camphorquinone (CQ) was used as photoinitiator, 0.9 wt% of 2-(dimethylamine)ethyl methacrylate (DMAEMA) as co-initiator, and 0.1 wt% of butylated hydroxytoluene (BHT) as the photopolymerization inhibitor. All reagents were purchased from Sigma-Aldrich. To prepare an experimental dental composite, silica Arsil (Zakłady Chemiczne Rudniki S.A., Rudniki, Poland) was silanized by 3-methacrylooxypropyltri-methoxysilane (γ-MPTS) from Unisil Sp. z o.o (Tarnów, Poland) according to the method described by Kleczewska J. et al. [33] and then hand-mixed in an agate mortar with a resin matrix to produce a composite filled with 45 wt%. To modify 5 g of the experimental dental composite, 0.5, 1.5, and 2.0 wt% of QASs—dimethyldioctadecylammonium bromide (DODAB or cetyltrimethylammonium bromide (CTAB)—were added to the resin matrix. Both salts were also obtained from Sigma-Aldrich. All reagents were of analytical grade and their details are described in Table 1 below. 

### 2.2. Samples Preparation

**Figure 3:**
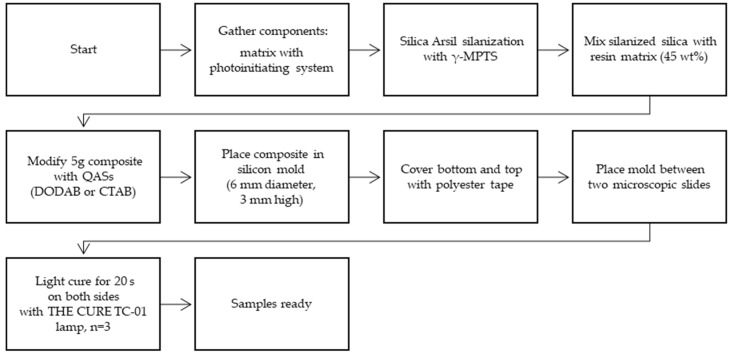
Sample preparation.

The experimental dental composite without or with addition of QAS was placed in a cylindrical silicon mold (6 mm diameter, 3 mm high), covered on the bottom and upper side with polyester tape (Hawe Striproll, Kerr, Bioggio, Switzerland) to prevent the formation of the oxide inhibition layer. To obtain even sample surfaces, mold with composite and polyester tape was placed between two microscopic slides, and the material was lightly cured for 20 s on both sides with THE CURE TC-01 polymerization lamp (Spring Health Products, Norristown, PA, USA). A flowchart of the sample preparation is presented below (Figure 3).

### 2.3. Antibacterial Properties Testing Methods

#### 2.3.1. Procedure for Surface Bactericidal Analysis with *Escherichia coli*, *Streptococcus mutans,* and *Candida albicans*

**Figure 4:**
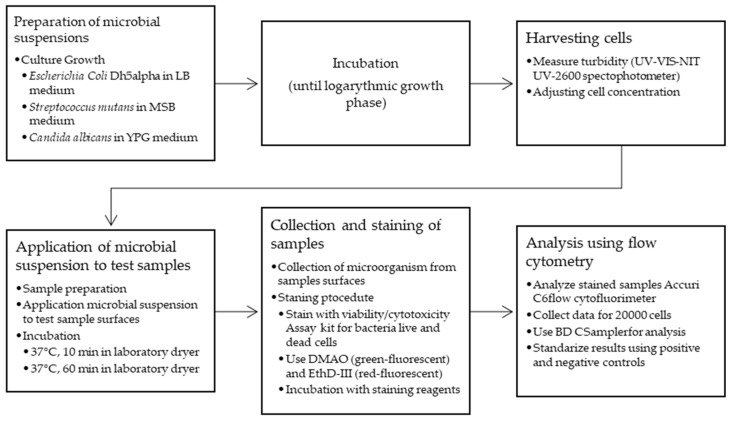
Biocidal properties study flowchart.

*Escherichia coli* DH5alpha, *Streptococcus mutans* NCTC 10449, *Candida albicans* NCCLS 11 (all from ATCC, Manassas, VA, USA) were used in this study. The bacterial or fungal suspensions (10 µL) were applied to the surfaces of the test sample, corresponding to the application of approximately 4 × 10^5^ cells in a logarithmic growth phase. Cell counts were determined by using a method previously developed in our lab. Simultaneous turbidance measurements using a UV-VIS-NIR UV-2600 spectrophotometer (Shimadzu, Kyoto, Japan), and cell counting using an automatic EVE NanoEntek cell counter (Seoul, Korea) enabled the development of a standardized method to determine cell abundance based on turbidimetric measurements. This method was standardized for each microorganism used separately and incubations were carried out for two contact times—10 min and 60 min at 37 °C in a laboratory dryer. After incubation, the bacterial suspension was carefully collected from the surface and then subjected to the staining procedure according to the ‘Viability/Cytotoxicity Assay kit for Bacteria Live and Dead Cells’ (ImmuniQ, Żory, Poland). After incubating with two fluorescent reagents (DMAO was a green-fluorescent nucleic acid dye that stained both live and dead bacteria and Ethidium Homodimer III (EthD-III) was a red-fluorescent nucleic acid dye that selectively stained dead bacteria with damaged cell membranes), the results were read using an Accuri C6 flow cytofluorimeter (BD Biosciences, Franklin Lakes, NJ, USA) and then analyzed using BD CSampler software (BD Biosciences, Franklin Lakes, NJ, USA). Simultaneous standardization was performed using a positive control (live cells) and a negative control (dead cells). Moreover, 20,000 passages were collected each time, corresponding to 20,000 cells analyzed. A suspension collected after 24 h of culture (logarithmic growth phase culture) was used. For the *Escherichia coli* strain DH5α, LB medium was prepared with the following composition: NaCl (1%), Bacto Peptone (1%), and yeast extract (0.5%), with a pH equal to 7.0. For *Candida albicans,* a YPG medium was used with the following composition: yeast extract (1%), Bacto Peptone (1%), and glucose (2%), with a pH of 7.4. For *Streptococcus mutans,* MSB medium (mitis-salivarius-bacitracin from BD Biosciences, Franklin Lakes, NJ, USA) was used [34]. The flow chart of the procedure is shown below (Figure 4). 

The results are presented as mean ± standard deviations (SDs). The results were analyzed using one-way ANOVA with a significance level of *p* < 0.05; for both 10 and 60 min of data, a post hoc HSD Tukey’s test was performed. Statistical analysis was performed using Microsoft Excel with Office 365.

#### 2.3.2. Bacterial/Yeast Surface Colonization

Three separate and independent tests were performed for each type of material, with two samples of a given material used in each test. For the surface susceptibility test of microbial colonization, each sample was photographed at a minimum of five random locations (in the center of the sample to eliminate the impact of counting disturbances caused by the sample periphery). A resin-based dental composite without quaternary ammonium salt addition was used as a control group for the study. The prepared samples were placed in a medium suitable for the species tested—an LB medium for *Escherichia coli*, an MSB medium for *Streptococcus mutans,* and a YPG medium for *Candida albicans*. Each pair of samples was prepared in two independent replicates. A standardized number of cells was introduced into 200 mL of culture medium prepared this way—the accepted standard is 1 mL of culture in a stationary growth phase with an absorbance at 680 nm equal to 1—this corresponds to approximately 2 × 10^3^ bacterial or yeast cells. The culture was incubated for 24 h at 37 °C. At the end of the incubation, the samples were sterilely removed and rinsed with sterile distilled water to remove unadhered cells. The sample’s surface was subjected to a bacterial or yeast cell counting procedure using a fluorescence microscope. The method is based on live/death staining with simultaneous use of two fluorescent dyes, namely, bisbenzimide and propidium iodide. The first one penetrates inside the bacteria/yeast and intercalates with the DNA, resulting in UV-stimulated luminescence, enabling visualization of live cells. The second also attaches to the DNA but does not penetrate the cell membrane, resulting in the visualization of dead cells. The study was conducted on a GX71 inverted-optics fluorescence microscope equipped with a DP 73 digital camera (Olympus, Kyoto, Japan). At least five images were taken for each sample at a random location (but reasonably in the center of the sample) [35]. The results are presented as mean ± standard deviations (SDs). The results were analyzed using one-way ANOVA with a significance level of *p* < 0.05, and the post hoc HSD Tukey’s test was performed. Statistical analysis was performed using Microsoft Excel with Office 365.

**Figure 5:**
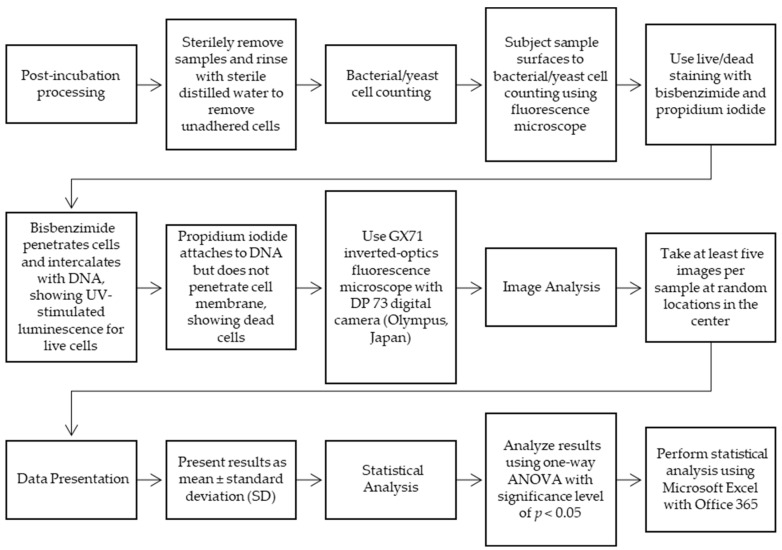
The scheme presented in the study of bacterial/yeast sample surface colonization.

The flowchart shown in Figure 5 presents the surface colonization of the bacterial/yeast samples.

## 3. Results

### 3.1. The Surface Bactericidal Analysis with Escherichia coli, Streptococcus mutans, and Candida albicans

**Figure A1:**
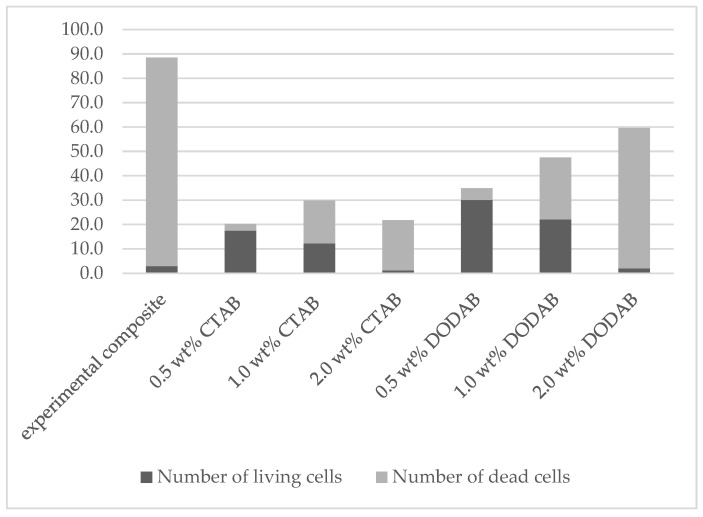
Influence of the amount of CTAB or DODAB on the number of living/dead cells of *Escherichia coli*.

**Figure A2:**
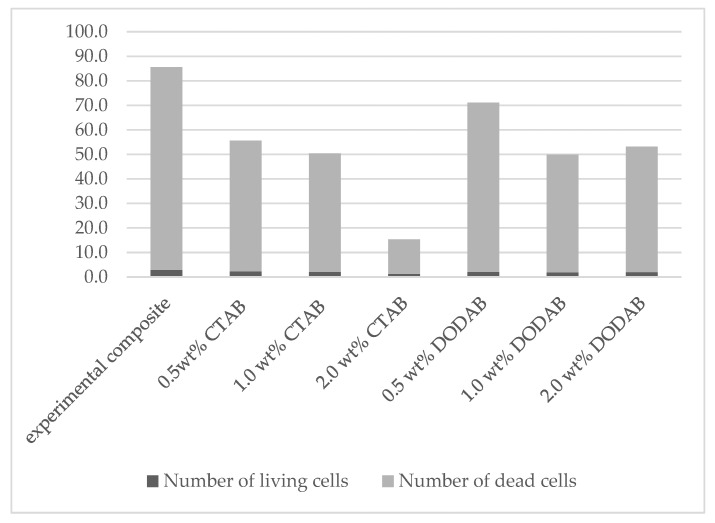
Influence of the amount of CTAB or DODAB on the number of living/dead cells of *Streptococcus mutans*.

**Figure A3:**
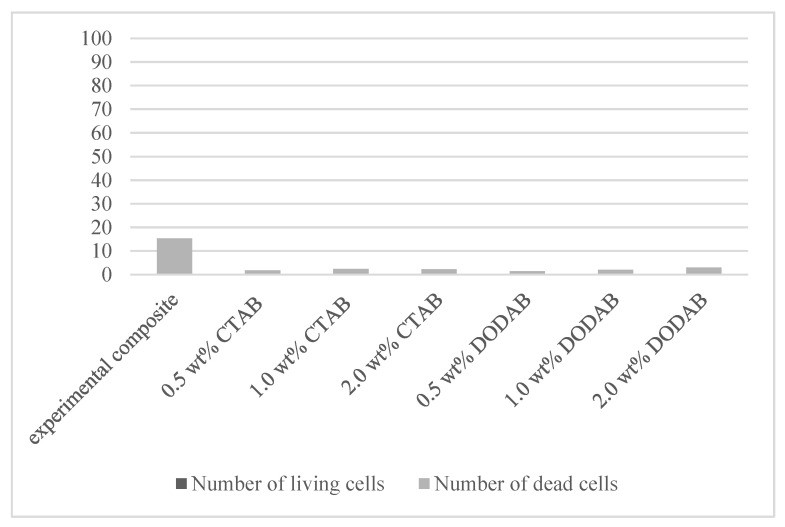
Influence of the amount of CTAB or DODAB on the number of living/dead cells of *Candida albicans*.

According to results shown in Table 2 (Figure A1, Figure A2 and Figure A3 and statistical data given in Appendix A—Table A1, Table A2, Table A3 and Table A4), CTAB and DODAB act as antibacterials. We can observe a positive correlation between increasing concentrations of quaternary ammonium salts and the ratio of dead cells in all kinds of tested microbes. *Streptococcus mutans* was the most sensitive pathogen in contact with the QAS-modified experimental dental composite. CTAB was more effective at causing the death of *Streptococcus mutans* than DODAB, especially in a concentration of 2.0 wt%—almost ¾ of Gram-positive bacteria were killed (73.9 wt%). In the case of Gram-negative bacteria (*Escherichia coli*), after both incubation times, CTAB was more effective in producing harmful effects than DODAB, and interactions with the surface were also stronger when the material contained a larger amount of QAS in its composition. Both salts killed over half of *Escherichia coli* cells after 60 min (2.0 wt% CTAB—65% dead cells, 2.0 wt% DODAB—54.5% dead cells). The most resistant pathogen in contact with QAS-modified surfaces was *Candida albicans*. This yeast seemed to be more sensitive when in contact with the 2.0 wt% CTAB-modified composite, where a slight difference (but not statistical significance) can be observed only after 60 min compared to DODAB at the same salt concentration. 

### 3.2. Antibacterial and Antifungal Properties of the Surface QAS-Modified Experimental Dental Composite

We assessed the susceptibility of microbial colonization on the surfaces of non-modified/modified experimental dental composites, as shown in Table 3 for *Escherichia coli*, Table 4 for *Streptococcus mutans,* and Table 5 for *Candida albicans*. With the increasing amount of QAS in the experimental dental composite, the average percentage of living cells dropped significantly from around 86% in the case of 0.5 wt% CTAB or DODAB to only a few percent when 2.0 wt% of the antimicrobial agent was added to dental material. There are no visible differences between the types of salt in contact with Gram-negative *Escherichia coli*. 

Even small concentrations of QAS in the experimental dental composite significantly influenced the average percentage of living *Streptococcus mutans* on sample surfaces. Almost all Gram-positive bacteria found on sample surfaces were dead. Only 3.0–8.5% of *Streptococcus mutans* cells were living on the assessed surfaces. DODAB was a more harmful agent against *Streptococcus mutans* when compared to CTAB, but both salts were very effective at preventing bacteria colonization on the sample surfaces.

As shown in Table 5, QAS additives slightly affected the colonization of experimental dental composites by *Candida albicans*. The yeasts were more resistant when in contact with modified materials compared to both types of bacteria mentioned earlier. Also, in this test, increasing the concentration of CTAB or DODAB promoted the prevention of *Candida albicans* colonization of the assessed surfaces. 

A graphical presentation of all results is available in Appendix A (Figure A1 and Figure A2).

## 4. Discussion

Quaternary ammonium compounds (QACs) are some of the most useful antimicrobial agents among the classical cationic surfactants [36]. Ishikawa S. et al. also confirmed that CTAB is an effective antimicrobial against *Escherichia coli*, but bacteria growth under anaerobiosis made cells resistant to cetyltrimethylammonium bromide [37]. So, in terms of the possible usage of this substance as an antimicrobial agent, it is important to provide further investigations to compare the mode of action of the CTAB- or DODAB-modified dental composite with or without contact with oxygen. Ribeiro R. et al. showed similar observations compared to ours. The addition of CTAB and DODAB to PMMA affected cell viability. Similar to our investigation, *Candida albicans* was less sensitive to contact with QAS than bacteria (*Escherichia coli* and *Staphylococcus aureus*) [38,39]. Leticia D. Melo et al. also observed that CTAB needed a smaller dose to act as an antimicrobial agent compared to DODAB, probably due to its higher diffusibility and appropriate hydrophobic-hydrophilic balance. It was also stated that longer alkyl chains in DODAB than in CTAB can result in a decrease in activity. Also, Makvandi P. et al. stated that incorporating QAC into resin-based composites has clinical importance because of the inhibition of oral bacteria and biofilm growth, and the best are QACs with 12–16 carbon atoms in chains [9,40]. In accordance with the literature, they also confirmed that Gram-positive bacteria like *Streptococcus mutans* and *Streptococcus aureus* are more sensitive to CTAB than Gram-negative bacteria. Gram-negative bacteria may change their outer envelope composition as a defense response to quaternary ammonium, so as shown in our results in Table 2, the percentage of dead cells of *Escherichia coli* is lower than *Streptococcus mutans* in the same group of tested materials. This difference in results may be caused by the above-mentioned mode of defense presented by Gram-negative bacteria [40]. As D.B. Viera and A.M. Carmona-Ribeiro reported in their paper, DODAB and CTAB have similar effects against *Candida albicans* [25]. Both salts, similar to our results, act only fungistatic during the first hour. As they mentioned, the antifungal effect of these QASs is not cell lysis, but the reversal of cell surface charge to the opposite (from negative to positive). A similar effect was reported in another publication, where DODAB was also more effective against *Candida albicans* than CTAB [40]. Cationic CTAB can form a micelle and does not disrupt the fungal cell membrane. Contrary to the paper, the bilayer-forming DODAB has been reported to be weaker against *Candida albicans* than CTAB [10]. This may be attributed to the fact that CTAB molecules can penetrate *Candida albicans* aggregates, but they cannot be reached by DODAB molecules. It is worth noting that it may also be caused by the molecular structures of DODAB and yeast cell aggregation as functions of cell concentration. DODAB cannot reach living cells inside cell aggregates. Also, the rigid gel state of DODAB may impede penetration into fungal cell walls and their cytoplasmic membranes or through the yeast aggregate/agglomerate structures [10]. CTAB is a good antibacterial and antistatic agent, and has biocidal activity against some Gram-negative bacteria and Gram-positive bacteria. DODAB salt decreases the viability of *Candida albicans*. The use of micromolar concentrations of QASs enables the death of bacteria; however, in the case of yeasts, much higher salt concentrations are needed [25]. 

## 5. Conclusions

To summarize—our study shows that experimental resin-based dental composites modified with CTAB or DODAB exhibit antibacterial and antifungal properties. A negative correlation was observed between increasing amounts of QAS in experimental materials and the viability of microorganisms. *Streptococcus mutans* are proven to be the most susceptible pathogens in contact with QAS, whereas *Candida albicans* displayed higher resistance to quaternary ammonium salts used in tests. A decrease in microbial colonization and an increase in dead cells suggest that even small amounts of CTAB or DODAB in the experimental dental composite may be effective in caries prevention. It is worth noting that composites modified with any of these salts acted quickly against these three types of microorganisms after only 10 min, and the duration of action increased their antimicrobial effectiveness. 

According to the obtained results, which confirm the biocidal activity of CTAB and DODAB incorporated into resin-based dental composited, and taking into account that the same modification of dental restorative material met the minimum requirements for the diametral tensile strength for these types of composites [4], further studies should focus on QAS-modified dental restorative material properties (i.e., use of different types and concentrations of QASs; modification of different types of dental restorative materials with QAS) to obtain material that may improve oral health by caries prevention. Based on our observation of a visibly smaller amount of living and dead cells on the surfaces of QAS-modified composites compared to unmodified material, microorganism adhesion to a dental composite surface needs to be analyzed. The surfactant character of QAS probably causes this phenomenon.

The studies presented here using *Escherichia coli*, *Streptococcus mutans,* and *Candida albicans* were preliminary studies to determine the initial antibacterial and antifungal properties of QAS-modified composites. As a next step, carrying out tests using caries-forming bacteria, such as *Lactobacillus acidophilus*, *Streptococcus salivarius,* and others, would be worthwhile. Moreover, the cytotoxicity of prepared composites with the addition of DODAB or CTAB has been assessed and is being prepared for publication.

## Figures and Tables

**Table 1:** Materials used to prepare the QAS-modified experimental dental composite.

Ingredient Name	Molecular Weight [g/mol]	Purity [%]	Ratio [wt%]	LOT/ Batch	Manufacturer
bisphenol A glycerolate dimethacrylate (bis-GMA)	512	>97	40	MIKCR9254	Sigma-Aldrich, St. Louis, MO, USA
diurethane dimethacrylate (UDMA)	470	>97	40	#MKCG8230	Sigma-Aldrich, Steinheim, Germany
2-hydroxyethyl methacrylate (HEMA)	130	>97	10	#GTBC3071V	Sigma-Aldrich, Steinheim, Germany
triethylene glycol dimethacrylate (TEGDMA)	470	>95	10	#STBH8825	Sigma-Aldrich, Steinheim, Germany
camphorquinone (CQ)	166	>97	0.4	09003AQV	Sigma-Aldrich, St. Louis, MO, USA
2-(dimethylamine)ethyl methacrylate (DMAEMA)	157	>98	0.9	#BCBZ6476	Sigma-Aldrich, Steinheim, Germany
butylated hydroxytoluene (BHT)	220	>99	0.1	#116K0036	Sigma-Aldrich, St. Louis, MO, USA
silica Arsil	<150 g/dm^3^	>95	45	260321	Zakłady Chemiczne Rudniki S.A., Rudniki, Poland
3-methacrylooxypropyltri-methoxysilane (γ-MPTS)	196	>95	-	20.10.2020	Unisil Sp. z o.o, Tarnów, Poland
dimethyldioctadecylammonium bromide (DODAB)	631	>98	0.5–2.0	BCBR19922V	Sigma-Aldrich, Steinheim, Germany
cetyltrimethylammonium bromide (CTAB)	364	>98	0.5–2.0	SLCH0757	Sigma-Aldrich, Product of China

**Table 2:** The analysis of the surface bactericide ability of materials, both non-modified and modified, with different concentrations of QAS after 10 and 60 min of incubation at 37 °C.

QAS [wt%]	Material	Time [min]	*Escherichia coli*	*Streptococcus mutans*	*Candida albicans*
			Dead Cells [%]	Dead Cells [%]	Dead Cells [%]
0.0	experimental composite	10	21.0	33.8	13.9
0.5	CTAB	10	19.4	35.0	10.2
1.0	CTAB	10	20.1	37.6	12.2
2.0	CTAB	10	37.2	40.6	15.4
0.5	DODAB	10	21.1	32.9	10.0
1.0	DODAB	10	22.7	35.6	10.5
2.0	DODAB	10	26.1	35.0	15.6
0.0	experimental composite	60	28.9	44.6	15.0
0.5	CTAB	60	18.8	42.9	13.0
1.0	CTAB	60	24.0	57.3	15.6
2.0	CTAB	60	65.0	73.9	23.9
0.5	DODAB	60	25.1	45.9	12.2
1.0	DODAB	60	35.8	54.7	15.6
2.0	DODAB	60	54.5	68.9	21.7

**Table 3:** *Escherichia coli* colonization of non-/QAS-modified experimental dental composites.

QAS [wt%]	Material	Number of Living Cells	Number of Dead Cells	Sum	Average Percent of Living Cells [%]
0.0	experimental composite	2.9 ± 1.0	85.6 ± 7.9	88.5 ± 8.2	3.3 ± 0.8
0.5	CTAB	17.5 ± 3.5	2.7 ± 1.2	20.2 ± 3.5	86.4 ± 5.9
1.0	CTAB	12.2 ± 3.2	17.7 ± 2.6	29.9 ± 3.3	40.6 ± 8.1
2.0	CTAB	1.2 ± 0.6	20.6 ± 3.9	21.8 ± 3.8	5.7 ± 2.9
0.5	DODAB	30.1 ± 4.3	4.8 ± 1.2	34.9 ± 4.8	86.2 ± 3.0
1.0	DODAB	22.1 ± 4.3	25.4 ± 3.9	47.5 ± 5.2	46.4 ± 6.9
2.0	DODAB	2.0 ± 1.1	57.6 ± 8.3	59.6 ± 8.6	3.3 ± 1.6

**Table 4:** *Streptococcus mutans* colonization of non-/QAS-modified experimental dental composites.

QAS [wt%]	Material	Number of Living Cells	Number of Dead Cells	Sum	Average Percent of Living Cells [%]
0.0	experimental composite	2.9 ± 1.0	82.7 ± 5.5	85.6 ± 6.0	3.4 ± 0.9
0.5	CTAB	2.3 ± 1.1	53.4 ± 5.9	55.7 ± 6.4	4.1 ± 1.8
1.0	CTAB	2.1 ± 0.7	48.4 ± 4.0	50.5 ± 4.2	4.1 ± 1.3
2.0	CTAB	1.2 ± 0.6	14.1 ± 4.4	15.3 ± 4.3	8.5 ± 4.7
0.5	DODAB	2.1 ± 0.7	69.0 ± 5.3	71.1 ± 5.3	3.0 ± 1.1
1.0	DODAB	1.9 ± 1.0	48.0 ± 6.8	49.9 ± 7.2	3.7 ± 1.8
2.0	DODAB	2.0 ± 1.1	51.2 ± 8.7	53.2 ± 8.8	3.8 ± 1.9

**Table 5:** *Candida albicans* colonization of non-/QAS-modified experimental dental composite.

QAS [wt%]	Material	Number of Living Cells	Number of Dead Cells	Sum	Average Percent of Living Cells [%]
0.0	experimental composite	50.9 ± 7.9	15.4 ± 2.8	66.3 ± 10.1	76.8 ± 2.2
0.5	CTAB	25.2 ± 2.8	1.8 ± 0.9	27.0 ± 2.4	93.2 ± 3.3
1.0	CTAB	21.4 ± 2.3	2.4 ± 0.8	23.8 ± 2.6	90.0 ± 3.2
2.0	CTAB	12.2 ± 4.8	2.3 ± 1.2	14.5 ± 4.8	82.7 ± 8.2
0.5	DODAB	31.3 ± 4.6	1.4 ± 0.5	32.7 ± 4.6	95.6 ± 1.8
1.0	DODAB	23.6 ± 3.2	2.1 ± 1.0	25.7 ± 2.8	91.6 ± 4.2
2.0	DODAB	17.1 ± 4.7	3.0 ± 1.3	20.1 ± 5.0	84.9 ± 7.1

## Data Availability

The raw data supporting the conclusions of this article will be made available by the authors upon request.

## Appendix A

**Table A1:** Statistical analysis of the surface bactericidal study with *Escherichia coli* after 10 min.

Group 1	Group 2	*p*-Value	Significance
0.5 wt% CTAB	1.0 wt% CTAB	0.7488	False
0.5 wt% CTAB	1.0 wt% DODAB	0.9896	False
0.5 wt% CTAB	2.0 wt% DODAB	0.0554	False
0.5 wt% CTAB	2.0 wt% CTAB	**0.0072**	**True**
0.5 wt% CTAB	experimental composite	**0.0001**	**True**
0.5 wt% CTAB	0.5 wt% DODAB	0.9632	False
0.5 wt% DODAB	1.0 wt% CTAB	0.3635	False
0.5 wt% DODAB	1.0 wt% DODAB	0.7081	False
0.5 wt% DODAB	2.0 wt% DODAB	**0.0057**	**True**
0.5 wt% DODAB	2.0 wt% CTAB	**0.0007**	**True**
0.5 wt% DODAB	experimental composite	**0.0001**	**True**
1.0 wt% CTAB	1.0 wt% DODAB	0.9875	False
1.0 wt% CTAB	2.0 wt% DODAB	0.4821	False
1.0 wt% CTAB	2.0 wt% CTAB	0.1234	False
1.0 wt% CTAB	experimental composite	**0.0006**	**True**
1.0 wt% DODAB	2.0 wt% DODAB	0.2227	False
1.0 wt% DODAB	2.0 wt% CTAB	**0.0412**	**True**
1.0 wt% DODAB	experimental composite	**0.0001**	**True**
2.0 wt% DODAB	2.0 wt% CTAB	0.9897	False
2.0 wt% DODAB	experimental composite	0.0596	False
2.0 wt% CTAB	Experimental composite	0.3201	False

**Table A2:** Statistical analysis of the surface bactericidal study with *Escherichia coli* after 60 min.

Group 1	Group 2	*p*-Value	Significance
0.5 wt% CTAB	0.5 wt% DODAB	**0.0000**	**True**
0.5 wt% CTAB	1.0 wt% CTAB	**0.0002**	**True**
0.5 wt% CTAB	1.0 wt% DODAB	**0.0000**	**True**
0.5 wt% CTAB	2.0 wt% CTAB	**0.0000**	**True**
0.5 wt% CTAB	2.0 wt% DODAB	**0.0000**	**True**
0.5 wt% CTAB	experimental composite	**0.0129**	**True**
0.5 wt% DODAB	1.0 wt% CTAB	**0.0002**	**True**
0.5 wt% DODAB	1.0 wt% DODAB	**0.0000**	**True**
0.5 wt% DODAB	2.0 wt% CTAB	**0.0000**	**True**
0.5 wt% DODAB	2.0 wt% DODAB	**0.0000**	**True**
0.5 wt% DODAB	experimental composite	**0.0129**	**True**
1.0 wt% CTAB	1.0 wt% DODAB	**0.0000**	**True**
1.0 wt% CTAB	2.0 wt% CTAB	**0.0000**	**True**
1.0 wt% CTAB	2.0 wt% DODAB	**0.0000**	**True**
1.0 wt% CTAB	experimental composite	**0.0001**	**True**
1.0 wt% DODAB	2.0 wt% CTAB	**0.0000**	**True**
1.0 wt% DODAB	2.0 wt% DODAB	**0.0000**	**True**
1.0 wt% DODAB	experimental composite	**0.0000**	**True**
2.0 wt% CTAB	2.0 wt% DODAB	**0.0000**	**True**
2.0 wt% CTAB	experimental composite	**0.0000**	**True**
2.0 wt% DODAB	experimental composite	**0.0000**	**True**

**Table A3:** Statistical analysis of the surface bactericidal study with *Streptococcus mutans* after 10 min.

Group 1	Group 2	*p*-Value	Significance
experimental composite	0.5 wt% DODAB	0.9971	False
experimental composite	1.0 wt% DODAB	0.3490	False
experimental composite	2.0 wt% DODAB	**0.0035**	**True**
experimental composite	0.5 wt% CTAB	0.9994	False
experimental composite	1.0 wt% CTAB	0.9432	False
experimental composite	2.0 wt% CTAB	0.9970	False
0.5 wt% DODAB	1.0 wt% DODAB	0.7601	False
0.5 wt% DODAB	2.0 wt% DODAB	**0.0282**	**True**
0.5 wt% DODAB	0.5 wt% CTAB	0.9047	False
0.5 wt% DODAB	1.0 wt% CTAB	0.9999	False
0.5 wt% DODAB	2.0 wt% CTAB	1.0000	False
1.0 wt% DODAB	2.0 wt% DODAB	0.6773	False
1.0 wt% DODAB	0.5 wt% CTAB	0.0806	False
1.0 wt% DODAB	1.0 wt% CTAB	0.9047	False
1.0 wt% DODAB	2.0 wt% CTAB	0.7611	False
2.0 wt% DODAB	0.5 wt% CTAB	**0.0005**	**True**
2.0 wt% DODAB	1.0 wt% CTAB	0.0570	False
2.0 wt% DODAB	2.0 wt% CTAB	**0.0285**	**True**
0.5 wt% CTAB	1.0 wt% CTAB	0.7815	False
0.5 wt% CTAB	2.0 wt% CTAB	0.9025	False
1.0 wt% CTAB	2.0 wt% CTAB	1.0000	False

**Table A4:** Statistical analysis of surface bactericidal study with *Streptococcus mutans* after 60 min.

Group 1	Group 2	*p*-Value	Significance
experimental composite	0.5 wt% DODAB	**0.0000**	**True**
experimental composite	1 wt% DODAB	**0.0000**	**True**
experimental composite	2 wt% DODAB	**0.0000**	**True**
experimental composite	0.5 wt% CTAB	**0.0000**	**True**
experimental composite	1.0 wt% CTAB	**0.0000**	**True**
experimental composite	2.0 wt% CTAB	**0.0000**	**True**
0.5 wt% DODAB	1.0 wt% DODAB	**0.0000**	**True**
0.5 wt% DODAB	2.0 wt% DODAB	**0.0000**	**True**
0.5 wt% DODAB	0.5 wt% CTAB	0.9968	False
0.5 wt% DODAB	1.0 wt% CTAB	**0.0205**	**True**
0.5 wt% DODAB	2.0 wt% CTAB	0.6323	False
1.0 wt% DODAB	2.0 wt% DODAB	1.0000	False
1.0 wt% DODAB	0.5 wt% CTAB	**0.0000**	**True**
1.0 wt% DODAB	1.0 wt% CTAB	**0.0000**	**True**
1.0 wt% DODAB	2.0 wt% CTAB	**0.0000**	**True**
2.0 wt% DODAB	0.5 wt% CTAB	**0.0000**	**True**
2.0 wt% DODAB	1.0 wt% CTAB	**0.0000**	**True**
2.0 wt% DODAB	2.0 wt% CTAB	**0.0000**	**True**
0.5 wt% CTAB	1.0 wt% CTAB	0.9162	False
0.5 wt% CTAB	2.0 wt% CTAB	0.8971	False
1.0 wt% CTAB	2.0 wt% CTAB	1.0000	False

**Table A5:** Statistical analysis of dead cell counts in *Escherichia coli* during colonization of experimental unmodified and modified composites.

Group 1	Group 2	*p*-Value	Significance
0.5 wt% CTAB	1.0 wt% CTAB	**0.001**	**True**
0.5 wt% CTAB	1.0 wt% DODAB	**0.001**	**True**
0.5 wt% CTAB	2.0 wt% DODAB	**0.001**	**True**
0.5 wt% CTAB	2.0 wt% CTAB	**0.001**	**True**
0.5 wt% CTAB	experimental composite	**0.001**	**True**
0.5 wt% CTAB	0.5 wt% DODAB	0.9	False
0.5 wt% DODAB	1.0 wt% CTAB	**0.001**	**True**
0.5 wt% DODAB	1.0 wt% DODAB	**0.001**	**True**
0.5 wt% DODAB	2.0 wt% DODAB	**0.001**	**True**
0.5 wt% DODAB	2.0 wt% CTAB	**0.001**	**True**
0.5 wt% DODAB	experimental composite	**0.001**	**True**
1.0 wt% CTAB	1.0 wt% DODAB	0.7334	False
1.0 wt% CTAB	2.0 wt% DODAB	**0.001**	**True**
1.0 wt% CTAB	2.0 wt% CTAB	**0.001**	**True**
1.0 wt% CTAB	experimental composite	**0.001**	**True**
1.0 wt% DODAB	2.0 wt% DODAB	**0.001**	**True**
1.0 wt% DODAB	2.0 wt% CTAB	**0.001**	**True**
1.0 wt% DODAB	experimental composite	**0.001**	**True**
2.0 wt% DODAB	2.0 wt% CTAB	0.9	False
2.0 wt% DODAB	experimental composite	0.9	False
2.0 wt% CTAB	experimental composite	0.9	False

**Table A6:** Statistical analysis of dead cell counts in *Streptococcus mutans* during colonization of experimental unmodified and modified composites.

Group 1	Group 2	*p*-Value	Significance
0.5 wt% CTAB	1 wt% CTAB	0.729	False
0.5 wt% CTAB	1 wt% DODAB	0.9	False
0.5 wt% CTAB	2 wt% DODAB	0.9	False
0.5 wt% CTAB	2 wt% CTAB	**0.0**	**True**
0.5 wt% CTAB	experimental composite	0.9	False
0.5 wt% CTAB	0.5 wt% DODAB	0.736	False
0.5 wt% DODAB	1.0 wt% CTAB	0.9	False
0.5 wt% DODAB	1.0 wt% DODAB	0.9	False
0.5 wt% DODAB	2.0 wt% DODAB	0.9	False
0.5 wt% DODAB	2.0 wt% CTAB	**0.0**	**True**
0.5 wt% DODAB	experimental composite	0.9	False
1.0 wt% CTAB	1.0 wt% DODAB	0.9	False
1.0 wt% CTAB	2.0 wt% DODAB	0.9	False
1.0 wt% CTAB	2.0 wt% CTAB	**0.0**	**True**
1.0 wt% CTAB	experimental composite	0.9	False
1.0 wt% DODAB	2.0 wt% DODAB	0.9	False
1.0 wt% DODAB	2.0 wt% CTAB	**0.0**	**True**
1.0 wt% DODAB	experimental composite	0.9	False
2.0 wt% DODAB	2.0 wt% CTAB	**0.0**	**True**
2.0 wt% DODAB	experimental composite	0.9	False
2.0 wt% CTAB	experimental composite	**0.0**	**True**

**Table A7:** Statistical analysis of dead cell counts in *Candida albicans* during colonization of experimental unmodified and modified composites.

Group 1	Group 2	*p*-Value	Significance
0.5 wt% CTAB	1.0 wt% CTAB	0.7488	False
0.5 wt% CTAB	1.0 wt% DODAB	0.9896	False
0.5 wt% CTAB	2.0 wt% DODAB	0.0554	False
0.5 wt% CTAB	2.0 wt% CTAB	**0.0072**	**True**
0.5 wt% CTAB	experimental composite	**0.0001**	**True**
0.5 wt% CTAB	0.5 wt% DODAB	0.9632	False
0.5 wt% DODAB	1.0 wt% CTAB	0.3635	False
0.5 wt% DODAB	1.0 wt% DODAB	0.7081	False
0.5 wt% DODAB	2.0 wt% DODAB	**0.0057**	**True**
0.5 wt% DODAB	2.0 wt% CTAB	**0.0007**	**True**
0.5 wt% DODAB	experimental composite	**0.0001**	**True**
1.0 wt% CTAB	1.0 wt% DODAB	0.9875	False
1.0 wt% CTAB	2.0 wt% DODAB	0.4821	False
1.0 wt% CTAB	2.0 wt% CTAB	0.1234	False
1.0 wt% CTAB	experimental composite	**0.0006**	**True**
1.0 wt% DODAB	2.0 wt% DODAB	0.2227	False
1.0 wt% DODAB	2.0 wt% CTAB	**0.0412**	**True**
1.0 wt% DODAB	experimental composite	**0.0001**	**True**
2.0 wt% DODAB	2.0 wt% CTAB	0.9897	False
2.0 wt% DODAB	experimental composite	0.0596	False
2.0 wt% CTAB	experimental composite	0.3201	False
